# β-arrestin-2-biased agonism of delta opioid receptors sensitizes transient receptor potential vanilloid type 1 (TRPV1) in primary sensory neurons

**DOI:** 10.1186/1744-8069-10-50

**Published:** 2014-08-01

**Authors:** Matthew P Rowan, Kalina Szteyn, Allison P Doyle, Ruben Gomez, Michael A Henry, Nathaniel A Jeske

**Affiliations:** 1 Departments of Oral and Maxillofacial Surgery, The University of Texas Health Science Center at San Antonio, MC 7908, 7703 Floyd Curl Drive, San Antonio, TX 78229-3900, USA; 2 Departments of Endodontics, The University of Texas Health Science Center at San Antonio, San Antonio, TX 78229, USA; 3 Departments of Pharmacology, The University of Texas Health Science Center at San Antonio, San Antonio, TX 78229, USA; 4 Departments of Physiology, The University of Texas Health Science Center at San Antonio, San Antonio, TX 78229, USA

**Keywords:** Chronic pain, Opioid, Allodynia, Hyperalgesia

## Abstract

Despite advances in understanding the signaling mechanisms involved in the development and maintenance of chronic pain, the pharmacologic treatment of chronic pain has seen little advancement. Agonists at the mu opioid receptor (MOPr) continue to be vital in the treatment of many forms of chronic pain, but side-effects limit their clinical utility and range from relatively mild, such as constipation, to major, such as addiction and dependence. Additionally, chronic activation of MOPr results in pain hypersensitivity known as opioid-induced hyperalgesia (OIH), and we have shown recently that recruitment of β-arrestin2 to MOPr, away from transient potential vanilloid eceptor type 1 (TRPV1) in primary sensory neurons contributes to this phenomenon. The delta opioid receptor (DOPr) has become a promising target for the treatment of chronic pain, but little is known about the effects of chronic activation of DOPr on nociceptor sensitivity and OIH. Here we report that chronic activation of DOPr by the DOPr-selective agonist, SNC80, results in the sensitization of TRPV1 and behavioral signs of OIH via β-arrestin2 recruitment to DOPr and away from TRPV1. Conversely, chronic treatment with ARM390, a DOPr-selective agonist that does not recruit β-arrestin2, neither sensitized TRPV1 nor produced OIH. Interestingly, the effect of SNC80 to sensitize TRPV1 is species-dependent, as rats developed OIH but mice did not. Taken together, the reported data identify a novel side-effect of chronic administration of β-arrestin2-biased DOPr agonists and highlight the importance of potential species-specific effects of DOPr agonists.

## Background

The management of chronic pain is a critical healthcare problem associated with soaring annual costs and immense physical and psychological burden to patients. Mild chronic pain can often be effectively controlled with acetaminophen or non-steroidal anti-inflammatory drugs. However, agonists acting at mu opioid receptors (MOPr) represent a mainstay treatment for moderate to severe chronic pain. Prolonged use of MOPr agonists can lead to severe side-effects, such as addiction, dependence, tolerance, constipation and respiratory depression, that limit their clinical utility [1]. Furthermore, chronic exposure to agonists at MOPr precipitates nociceptive hypersensitivity known as opioid-induced hyperalgesia (OIH) [2]. Although OIH has been well characterized in animal models [3-14] and clinical reviews/reports [8,15-19], OIH treatment in patients is difficult [19], in part because increasing opioid doses exacerbate OIH symptoms. The mechanisms underlying development and maintenance of OIH appear to have complicated central [6,16,20] and peripheral [13,21] components, but the transient receptor potential vanilloid type 1 receptor (TRPV1) appears to play a key role in the development and/or maintenance of OIH [13], especially in primary afferent neurons [21].

TRPV1 is a nonselective ligand-gated ion channel that is activated by heat, acid, and chemical agonists, including the selective agonist capsaicin [22-24]. TRPV1 is subject to dynamic regulation, mainly via phosphorylation (sensitization) and dephosphorylation (desensitization) by a several kinases [25]. Additionally, recent studies identified that the arrestin family of proteins, namely β-arrestin2, dynamically regulates the activity of several TRP channels [26,27], including TRPV1 [21,28]. Specifically, β-arrestin2 associates with TRPV1 and scaffolds phosphodiesterase PDE4D5, which decreases cyclic AMP levels leading to the desensitization of TRPV1 via reduced protein kinase A (PKA) activity [28]. We have recently demonstrated that chronic activation of peripheral MOPr with β-arrestin2-biased MOPr agonists leads to recruitment of β-arrestin2 to MOPr, a reduced association between β-arrestin2 and TRPV1 and a resulting sensitization of TRPV1 in primary sensory neurons, precipitating behavioral symptoms of OIH [21].

Since few other options exist that are safe and effective for the treatment of chronic pain, new and innovative approaches are needed to identify novel targets. Delta opioid receptors (DOPr) are gaining considerable attention as promising new targets in the treatment of chronic pain [29-32]. DOPr is a seven transmembrane spanning receptor, often termed G protein-coupled receptor (GPCR), that is expressed in the central and peripheral nervous system and inhibits nociceptive signaling when activated by agonists [30]. DOPr agonists are antinociceptive in behavioral models [29,30] and are highly efficacious at reducing chronic pain with reduced tolerance and dependence when compared to MOPr agonists [33-36]. Furthermore, DOPr agonists have shown promise as potential treatments for depression [37], which is often comorbid with chronic pain [38]. Due to their reduced side-effect profile, efficacy during chronic treatment, and potential efficacy with comorbid disorders, DOPr agonists hold great promise as novel therapeutics for the treatment of pain. However, few have studied side-effects of chronic DOPr activation or evaluated whether DOPr agonists are capable of producing OIH following chronic administration. Here we show that, like MOPr agonists, chronic administration of β-arrestin2-biased agonists of DOPr also sensitizes TRPV1 on primary sensory neurons and leads to the development of behavioral symptoms of OIH in rats. Interestingly, β-arrestin2 cross-talk with DOR/TRPV1 is species dependent, as DOPr agonists failed to sensitize TRPV1 or produce behavioral symptoms of OIH in mice or in primary sensory neurons from mice.

## Results

### DOPr and TRPV1 are co-localized on peptidergic and nonpeptidergic sensory neurons

**Figure 1:**
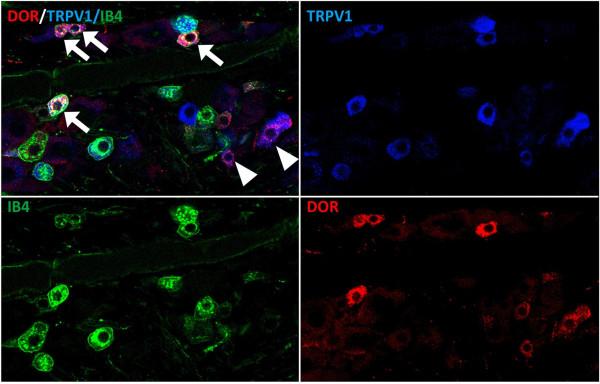
**DOPr co-expression with TRV1 in Trigeminal Ganglia Neurons.** Confocal micrograph shows DOPr (DOR, red) expression in a subset of sensory neurons in the rat trigeminal ganglion that includes sensory neurons with both (arrows) IB4 (green) and TRPV1 (blue) or with TRPV1 alone (arrowheads).

Studies in mice demonstrate that DOPr and TRPV1 do not colocalize in sensory neurons of the dorsal root ganglia (DRG) and that DOPr is found primarily in nonpeptidergic, small-diameter neurons that give rise to unmyelinated axons [39]. For this reason, it was proposed that DOPr agonists affect only mechanical, not thermal, nociception [40], but this is in contrast to the established role that DOPr agonists have on thermal allodynia in rats at both spinal [41] and peripheral locations [42-45]. Additionally, DOPr and TRPV1 colocalize in rat dental pulp [46] and primary afferent neurons of the rat DRG [47], with DOPr present in both peptidergic and nonpeptidergic DRG neurons [47]. Since sensory neurons from the rat trigeminal ganglia (TG) were used for *in vitro* studies, we qualitatively evaluated DOPr and TRPV1 expression relationships in rat TG tissue sections with immunohistochemistry and confocal microscopy. Results demonstrate DOPr colocalization with TRPV1 in a subpopulation of both nonpeptidergic/IB4+ (arrows) and IB4- (arrowheads) sensory neurons (Figure 1). The extent of colocalization was not quantified, but it was estimated that roughly 40% of all neurons expressed TRPV1, 40% expressed DOPr, and approximately half of the cells that were positive for TRPV1 were also positive for DOPr (Figure 1). These data support the hypothesis that DOPr activation could directly affect TRPV1 channel activity and/or sensitivity to a stimulus.

### Pretreatment with SNC80, not ARM390, recruits β-arrestin2 to DOR, away from TRPV1, in primary sensory neurons

**Figure 2:**
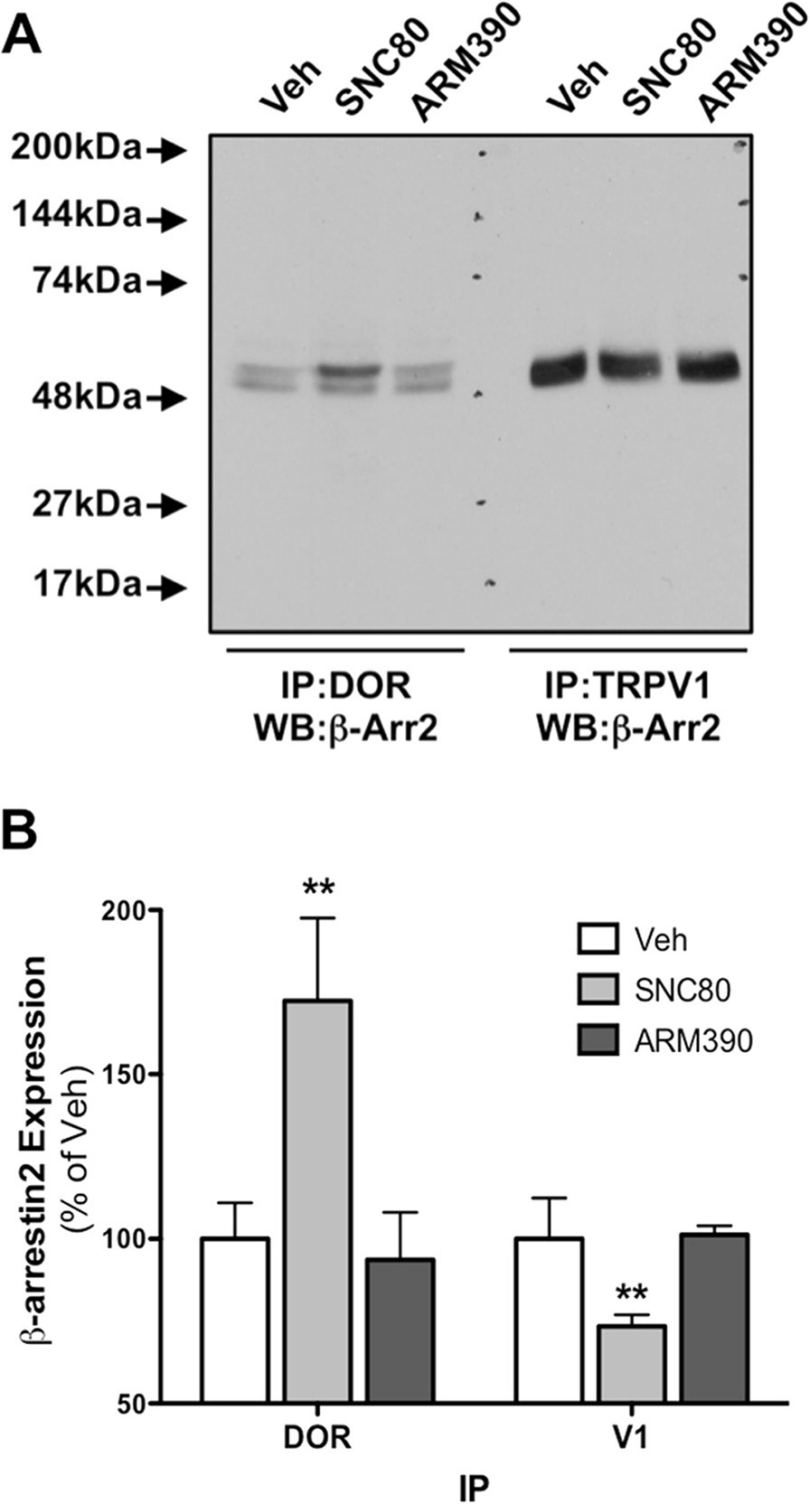
**β-arrestin2 is recruited to DOPr by SNC80, not ARM390, in sensory neurons.** TG neurons from rats were pretreated with SNC80 (1 μM), ARM390 (1 μM), or vehicle (0.1% DMSO) for 30 min. Cells were rinsed and association of DOPr or TRPV1 with β-arrestin2 was assessed via co-immunoprecipitation and Western blot. **A)** Representative blot following IP for DOPr or TRPV1 and probe for β-arrestin2. **B)** Mean ± SEM from 3 independent experiments per treatment condition. **, p > 0.01 by one-way ANOVA.

Our previous work with MOPr demonstrated that β-arrestin2-biased agonists differentially recruit β-arrestin2 to MOPr and away from TRPV1 [21]. Given that DOPr and TRPV1 are coexpressed in rat sensory neurons (Figure 1) and MOPr and DOPr signal through similar effector mechanisms with respect to β-arrestin2 [30,48-52], it follows that activation of DOPr with ligands that recruit β-arrestin2 would also reduce β-arrestin2 association with TRPV1. To test this, rat TG neurons were treated with DOPr-selective agonists, SNC80 (1 μM, 30 min) or ARM390 (1 μM, 30 min) and the association of β-arrestin2 with DOPr or TRPV1 was evaluated by coimmunoprecipitation. SNC80 and ARM390 are structurally similar and have similar affinities and efficacies for DOPr activation and inhibition of allodynia [53-55]. However, SNC80 effectively recruits β-arrestin2 to DOPr, while ARM390 does not [53,55-57]. Following treatment with SNC80 there was a significant increase in the association of β-arrestin2 and DOPr and a significant decrease in the association of β-arrestin2 and TRPV1 (Figure 2). Consistent with earlier reports on biased agonist activity [49,54-57], ARM390 did not recruit β-arrestin2 to DOPr and did not affect the association of β-arrestin2 and TRPV1 (Figure 2).

### Pretreatment with SNC80, not ARM390, produces species-dependent TRPV1 sensitization

**Figure 3:**
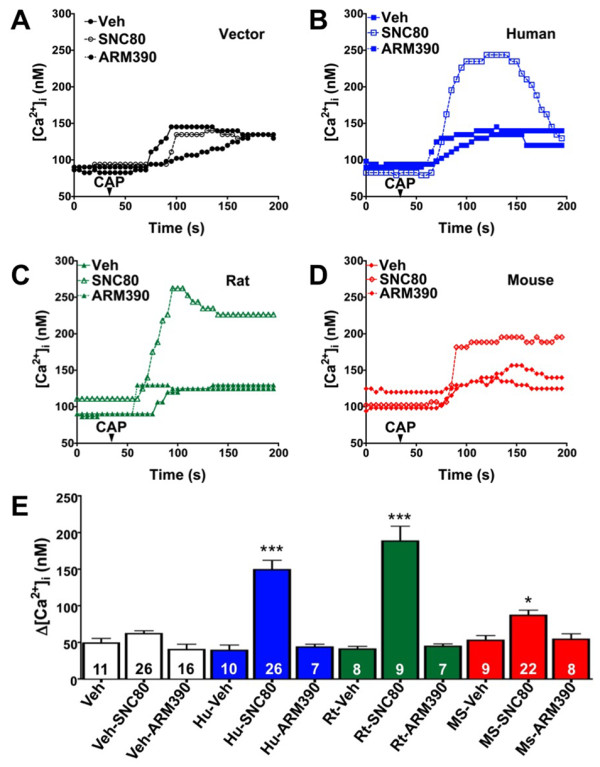
**SNC80, not ARM390, sensitizes TRPV1 in primary sensory neurons.** TG neurons from rats were nucleofected with empty vector (pEYFP-N1, **A)**, human **(B)**, rat **(C)**, or mouse **(D)** DOPr-YFP cDNA (Hu, Rt, Ms, respectively) and treated with SNC80 (1 μM, 30 min), ARM390 (1 μM, 30 min), or vehicle (0.1% DMSO). Real-time calcium responses from individual YFP-positive cells were measured before and after exposure to capsaicin (CAP, 50 nM) and the net change in intracellular calcium accumulation (Δ[Ca^2+^]_i_) was determined. Representative traces **(A-D)** and the mean ± SEM **(E)** of the difference in pre- and post-CAP response for the number of cells indicated at the bottom of each bar **(E)**. ***, *p > 0.001, 0.05 by one-way ANOVA.

**Figure 4:**
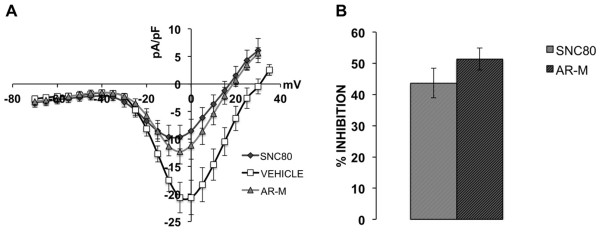
**SNC80 and ARM390 are equally effective at inhibiting N-type voltage-gated calcium currents in primary sensory neurons.** TG neurons from rats were nucleofected with rat DOPr-YFP cDNA and N-type voltage-gated calcium currents were recorded 24–48 h after isolation. Maximal current amplitudes were observed between −10 and −5 mV. Control currents were recorded first in SES with 0.1% DMSO (Vehicle). Following bath application of SNC80 (1 μM, 5 min) currents were again recorded. Cells that responded to SNC80 were washed with SES (5 min) and currents were recorded again prior to bath application of ARM390 (1 μM, 5 min) and final current measurement. Representative single cell current–voltage curves **(A)** and the mean ± SEM extent of current inhibition (expressed as % inhibition) for 10 and 12 cells for SNC80 and ARM390, respectively **(B)**.

β-arrestin2 desensitizes TRPV1 [28] and recruitment of β-arrestin2 away from TRPV1 with chronic administration of MOPr agonists results in sensitized TRPV1 and behavioral symptoms of OIH [21]. Treatment of rat sensory neurons with SNC80 results in the recruitment of β-arrestin2 to DOPr, away from TRPV1 (Figure 2), but the functional significance remains unclear. To evaluate this *in vitro* we treated cultured sensory neurons with SNC80 (1 μM, 30 min) or ARM390 (1 μM, 30 min) prior to exposure to the TRPV1 agonist, capsaicin (CAP, 50 nM), and monitored intracellular calcium accumulation as a response to TRPV1 activation, similar to previous studies [21,28,58-61]. To identify positive DOPr expression, rat neurons were nucleofected with YFP-labeled rat DOPr and recordings were taken from YFP-positive cells only. Pretreatment of sensory neurons with SNC80 significantly enhanced responses to CAP, whereas ARM390 had no effect (Figure 3C). TRPV1 was also significantly sensitized in sensory neurons nucleofected with YFP-labeled human DOPr (Figure 3B) following SNC80 pretreatment compared to ARM390 (Figure 3). However, in sensory neurons nucleofected with YFP-labeled mouse DOPr (Figure 3D), SNC80 sensitization of TRPV1 responses was significantly less than that seen for rat-DOPr- or human-DOPr-nucleofected neurons (Figure 3). Nucleofection efficiency (approximately 10-20%) was similar for rat, human, and mouse DOPr cDNAs. The concentrations of SNC80 and ARM390 chosen for the current study were validated in previous reports [55,57], but to verify that the concentrations of SNC80 and ARM390 were effective in our culture system, we evaluated the ability of SNC80 and ARM390 to acutely inhibit N-type voltage-gated calcium channels (VGCC), as we have before [21]. Application of SNC80 (1 μM, 5 min) or ARM390 (1 μM, 5 min) inhibited N-type VGCC by 44 ± 5 and 51 ± 4%, respectively (Figure 4).

### Chronic administration of SNC80, not ARM390, produces behavioral symptoms of OIH in rats, not mice

**Figure 5:**
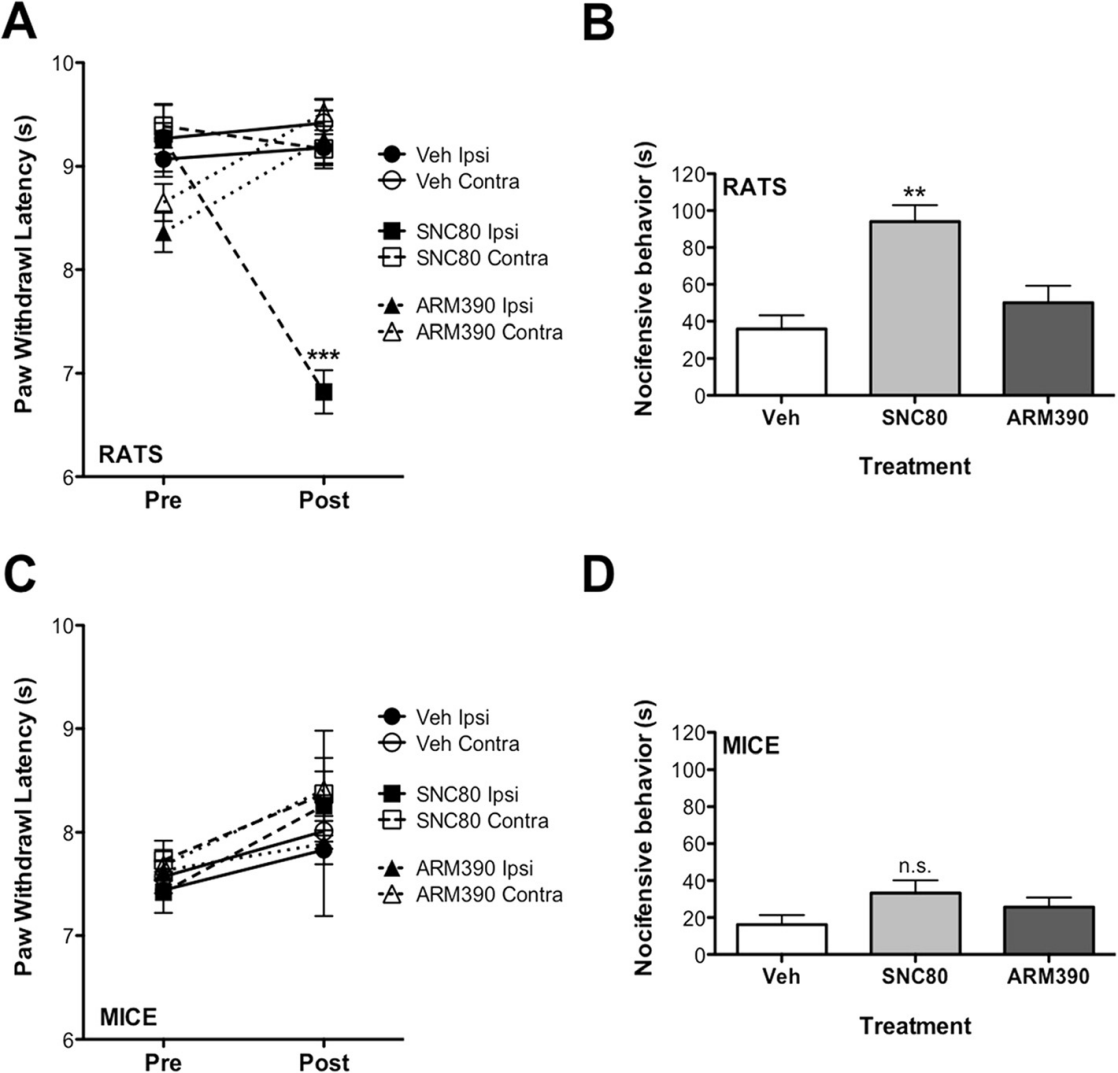
**Chronic administration of SNC80, not ARM390, results in behavioral symptoms of opioid-induced hyperalgesia in rats, not mice. A & C)** Following baseline thermal testing (Pre), separate groups of rats **(A)** and mice **(C)** received intraplantar injections once daily for 5 days with SNC80 (20 μg rats, 10 μg mice), ARM390 (20 μg rats, 10 μg mice), or vehicle (4% DMSO, 2% Tween20, PBS). On day 6 (Post), animals were re-tested for thermal sensitivity at least 24 h after the last injection. ***p > 0.001 by two-way ANOVA. **B & D)** Following thermal testing on day 6, animals received an intraplantar injection of capsaicin (0.5 μg rats, 0.1 μg mice) and nocifensive behavior was quantified for 5 min. **p > 0.01 by one-way ANOVA. n.s. = not significant.

**Figure 6:**
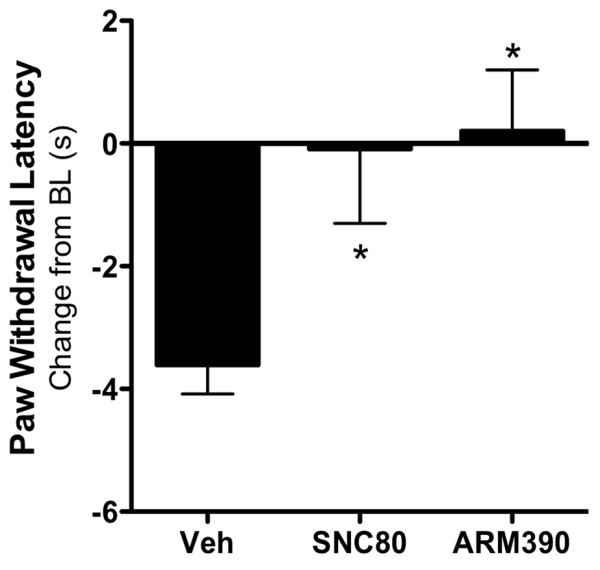
**Acutely administered SNC80 and ARM390 are equally effective at blocking thermal allodynia in rats.** Following baseline thermal testing separate groups of rats received intraplantar bradykinin (25 μg) 15 minutes before co-injection of prostaglandin E2 (300 ng) and SNC80 (20 μg), ARM390 (20 μg), or vehicle (4% DMSO, 2% Tween20, PBS). Paw withdrawal latency was measured after 20 min, and data are expressed as the mean ± SEM change from individual pre-injection baseline response for each animal. n = 6 rats per group. *, p > 0.05 by one-way ANOVA.

To determine the physiological significance of DOPr-mediated sensitization of TRPV1, intraplantar injections were performed once daily for five days with SNC80 (20 μg) or ARM390 (20 μg). Administering daily opioid injections is a common model of OIH in animals [7] and has been used with peripheral injections [21] to evaluate the effects of chronic opioid administration directly on primary sensory neurons and avoid confounding central mechanisms. Rats injected chronically with SNC80, not ARM390, displayed a significant reduction in thermal sensitivity (Figure 5A) and a significant increase in the sensitivity to CAP (Figure 5B). Alternatively, SNC80 induced neither thermal (Figure 5C) nor CAP (Figure 5D) sensitivity in mice following chronic injections, consistent with reduced measures of TRPV1 response sensitization (Figure 3). The doses of SNC80 and ARM390 chosen for these studies have been validated by others [55] and were equally effective at inhibiting thermal allodynia when administered acutely (Figure 6).

## Discussion

Chronic administration of agonists at MOPr produces OIH, which limits the clinical efficacy of opioids in the treatment of chronic pain [2]. Furthermore, OIH is difficult to treat, as increasing opioid dose results in a further exaggeration of pain symptoms [19]. Agonists at DOPr hold promise in the treatment of chronic pain [29,32], but a potential role for DOPr agonists in the development of OIH has not been evaluated. Understanding DOPr-mediated OIH and the potential mechanisms involved is critical to developing effective DOPr agonists for the treatment of chronic pain.

This is the first demonstration of chronic administration of DOPr agonists producing cellular and behavioral characteristics of OIH. Results presented here demonstrate that β-arrestin2 is recruited to DOPr, thereby decreasing TRPV1/β-arrestin2 association. Furthermore, TRPV1 sensitization is biased-agonist- and species-dependent, as cells expressing human or rat DOR display significantly greater enhancement than cells expressing mouse DOR. Furthermore, chronic administration of DOR agonists *in vivo* produces behavioral signs of OIH in rats, not mice, supporting the concept that DOR-TRPV1 cross-talk is species-dependent.

The location and function of DOPr on primary sensory neurons remains controversial. Some have shown that DOPr is not expressed in TRPV1-positive DRG sensory neurons in genetically-modified mice [39] and contend that DOPr agonists only inhibit mechanical, not thermal, nociception [40]. Others have demonstrated that DOPr agonists effectively inhibit thermal allodynia in rats when administered spinally [41] or peripherally [42-45]. Additionally, DOPr and TRPV1 colocalize in rat dental pulp [46] and primary afferent neurons of the rat DRG [47], with DOPr present in both peptidergic and nonpeptidergic DRG neurons [47]. Taken together with immunohistochemical results presented here in rats (Figure 1), it is clear that the distribution and function of DOPr is different in mice and rats, and presumably humans (Figure 3), and caution is advised when drawing broad conclusions about the function of DOPr across multiple species. Furthermore, clinical and preclinical trials evaluating DOPr agonists as novel analgesics should consider the use of different species whenever possible.The differential sensitization of TRPV1 by DOPr from rat, human, or mouse origin (Figure 3) suggests a functional difference from unique amino acid sequence, not a difference in localization or expression, as nucleofection efficiency was similar regardless of cDNA origin. Furthermore, the same cells were used for the investigation of DOPr species differences to control for the expression of β-arrestin2 and TRPV1. However, amino acid sequence comparison would likely serve a valuable role in the identification of species-unique differences that govern agonist effects, both within the receptor itself and within cross-signaling platforms such as β-arrestin paradigms highlighted in these studies. While acute inhibition of N-type VGCC was used as a control experiment to verify that SNC80 and ARM390 were equally effective in our culture system, kinetic studies would also be valuable to determine if differences manifest following prolonged pretreatment times, due to kinetic differences in β-arrestin2 recruitment.

DOPr agonists are effective in the treatment of chronic pain across a variety of preclinical models [29,30] and demonstrate reduced tolerance and dependence when compared to MOPr agonists [33-36]. Chronic pain is often comorbid with psychological disorders, such as depression [38], and DOPr agonists have demonstrated efficacy in animal models of depression [37]. For these reasons it is tempting to speculate broad use for DOPr agonists as future analgesics, but caution must again be exercised. DOPr agonists also have the potential to develop side-effects, such as seizures, that may limit clinical efficacy [32]. Unlike MOPr, DOPr undergoes unique trafficking [34,36,57], which may decrease the broad application of DOPr agonists for the treatment of chronic pain. However, due to their overall reduced side-effect profile and continued efficacy during chronic treatments in preclinical models, DOPr agonists hold promise as novel therapeutics for the treatment of chronic pain. In our previous study [21], we demonstrated that agonists at MOPr that did not recruit β-arrestin2 neither sensitized TRPV1 nor produced symptoms of OIH, and may therefore hold clinical promise. However, ligands that activate MOPr would still be expected to produce tolerance and dependence, so DOPr agonists might be better suited for the treatment of chronic pain. Results presented here highlight that DOPr agonists that do not recruit β-arrestin2 merit further investigation as novel therapeutics for the treatment of chronic pain for their potential to effectively manage chronic pain without inducing DOPr-mediated OIH. Future clinical and preclinical trials should consider the use of different species when evaluating DOPr agonists as novel analgesics.

## Methods

### Animals

All procedures using animals were approved by the Institutional Animal Care and Use Committee of The University of Texas Health Science Center at San Antonio and were conducted in accordance with policies for the ethical treatment of animals established by the National Institutes of Health and International Association for the Study of Pain. Male C57BL6 mice (22–25 g) and male Sprague–Dawley rats (175–200 g) used in these studies were from Charles River (Wilmington, MA).

## Materials

(+)-4-[(α*R*)-α-((2*S*,5*R*)-4-Allyl-2,5-dimethyl-1-piperazinyl)-3-methoxybenzyl]-*N*,*N*-diethylbenzamide (SNC80) and *N*,*N*-Diethyl-4-(phenyl-4-piperidinylidenemethyl)-benzamide hydrochloride (ARM390) were purchased from Tocris. All tissue culture reagents and media were from Invitrogen, and all other drugs and chemicals were from Sigma Aldrich unless otherwise indicated.

### Behavior

All injections were given intraplantarly in 50 μl (rat) or 10 μl (mouse) volumes via a 28-gauge needle inserted through the lateral footpad just under the skin to minimize tissue damage. Drug stocks were dissolved in PBS, or PBS with 2% Tween20 (for experiments with DMSO). Paw withdrawal latency to a thermal stimulus was measured by blinded observers with a plantar test apparatus (IITC, Woodland Hills, CA) as previously described [62]. Nocifensive behavior in response to CAP (Tocris Bioscience, Minneapolis, MN; 0.5 μg and 0.1 μg for rats and mice, respectively) was defined as hindpaw lifting, flinching, or licking and was quantified by blinded observers for 5 min as previously described [21,61].

### Primary TG neuron culture

Rat TG neurons were cultured as described previously [21]. Briefly, rats were sacrificed by decapitation, TG neurons were dissociated and digested with collagenase/trypsin, cells were centrifuged, enzymes were aspirated, and the cell pellet was re-suspended in Dulbecco’s modified Eagle’s medium supplemented with 10% fetal bovine serum, 100 ng/ml nerve growth factor (Harlan), 1% penicillin/streptomycin and 1% glutamine, then placed on poly-D-lysine coated 10 cm plates (BD; co-immunoprecipitation), or poly-D-lysine- and laminin-coated coverslips (BD; electrophysiology, calcium imaging). Cultures were maintained at 37**°**C, 5% CO_2_ for 5–7 d for co-immunoprecipitation experiments and 1–2 d for calcium imaging and electrophysiology.

### Nucleofection

For calcium imaging and electrophysiology experiments, TG neurons were isolated as described above and nucleofected (Lonza) as described previously [21,63,64] prior to plating on poly-D-lysine/laminin-coated coverslips. Cells were imaged 48–72 h following plating. Rat and mouse DOPr-YFP cDNAs were a generous gift from Dr. Nigel Bunnett (Monash University). Human DOPr cDNA was purchased from Missouri S&T (OPRD1) and cloned into pEYFP-N1 (Clontech).

### Immunohistochemistry

The TGs from a male Sprague Dawley rat were obtained, processed and stained as previously described [65,66] with IB4 isolectin from *Griffonia simplicifolia* labeled with Alex Fluor 488 conjugate (Molecular Probes I21411 at 1:1000 dilution), and rabbit anti-rat DOPr (Millipore AB5503 at 1:250 dilution) and guinea pig anti-rat TRPV1 (Neuromics GP14100 at 1:2000 dilution) polyclonal antibodies. Species-appropriate Alexa Fluor secondary antibodies (Molecular Probes at 1:200 dilution) were used to visualize DOPr (Alexa Fluor 568) and TRPV1 (Alexa Fluor 633). Immunofluorescence was evaluated and images were obtained with the use of a Nikon 90i microscope equipped with a C1si laser scanning confocal imaging system (Nikon, Melville, NY). Images were processed for illustration purposes with Adobe Photoshop CS2 (Adobe , San Jose, CA).

### Co-immunoprecipitation

TG neuron cultures were pretreated as indicated, harvested by scraping, homogenized, and plasma membranes were isolated by centrifugation. Total protein was quantified using the Bradford assay, and equal amounts of protein from each treatment were immunoprecipitated and analyzed as described previously [21] using antibodies to TRPV1 (Santa Cruz, R130), β-arrestin2 (Santa Cruz, H9) or DOPr (Abcam, ab66317). Antibody efficacy and specificity were verified by the manufacturers and BLAST sequence analysis, and used in previous publications [21,28,58,60,67].

### Calcium imaging

Changes in intracellular calcium concentration were measured as described previously [21]. Briefly, TG neurons cultured on poly-D-lysine-coated coverslips were incubated with Fura-2 AM (2 μM, 30 min, 37°C) in the presence of 0.05% Pluronic (EMD Millipore) in a standard extracellular solution (SES) containing (in mM): 140 NaCl, 5 KCl, 2 CaCl_2_, 1 MgCl_2_, 10 D-glucose, pH 7.4. Nucleofected cells were identified by the presence of YFP.

### Electrophysiology

Opioid-mediated inhibition of N-type VGCC was measured as described [67]. Briefly, patch clamp capillaries (2–4 MΩ) were pulled from borosilicate glass (World Precision Instruments, Sarasota, FL) with a micropipette puller (Narishige, Japan). Currents were measured in whole-cell configuration with an EPC-10 amplifier (HEKA, Germany) and analyzed with Patch Master software (HEKA). Giga seal and whole-cell configuration were established in SES containing (in mM): 145 NaCl, 5 KCl, 2 MgCl_2_, 2CaCl_2_, 10 HEPES, and 10 glucose (pH 7.4), then changed to extracellular solution containing (in mM): 145 NMDG-Cl, 2 MgCl_2_, 3 BaCl_2_, 10 HEPES, and 10 glucose (pH 7.4). Pipette solution contained (in mM): 120 CsCl, 1 MgCl_2_, 10 EGTA, 10 HEPES, 4 Mg-ATP, 0.3 Na-GTP (pH 7.2). The voltage-gated Ca^2+^currents were activated by pulses from −70 to 25 mV (150 ms, 5 mV steps, 5 s intervals) from a holding potential of −70 mV. SNC80 (1 μM) and ARM390 (1 μM) were applied via bath application and only cells that showed reversible effects of drug treatment were included in analysis. The identity of the currents was verified with application of the N-type calcium channel inhibitor α-conotoxin (Alomone Labs, Israel).

## Abbreviations

DOPr: Delta opioid receptor; IB4: Isolectin B4; MOPr: Mu opioid receptor; OIH: Opioid-induced hyperalgesia; PBS-Tw: Phosphate buffered saline-Tween; PKC: Protein kinase A; TRPV1: Transient receptor potential family V1; VGCC: N-type voltage-gated calcium channels; YFP: Yellow fluorescent protein.

## Competing interests

The authors declare that they have no competing interests.

## Authors’ contributions

Participated in research design: MPR, MAH, NAJ. Conducted experiments: MPR, KS, AD, RG, MAH, NAJ. Performed data analysis: MPR, KS, MAH, NAJ. Contributed to the writing of the manuscript: MPR, KS, MAH, NAJ. All authors read and approved the final manuscript.
